# Dissipation of mesoscale eddies at a western boundary via a direct energy cascade

**DOI:** 10.1038/s41598-022-05002-7

**Published:** 2022-01-18

**Authors:** D. Gwyn Evans, Eleanor Frajka-Williams, Alberto C. Naveira Garabato

**Affiliations:** 1 National Oceanography Centre, Southampton, UK; 2 Ocean and Earth Science, University of Southampton, Southampton, UK

**Keywords:** Ocean sciences, Physical oceanography

## Abstract

The fate of mesoscale eddy kinetic energy represents a large source of uncertainty in the global ocean energy budget. Satellite altimetry suggests that mesoscale eddies vanish at ocean western boundaries. However, the fate of the eddies’ kinetic energy remains poorly known. Here we show that the generation of small-scale turbulence as eddy flow impinges on the steep and corrugated slope of an ocean western boundary plays a dominant role in the regional decay of mesoscale eddy kinetic energy. We compare altimetry-based estimates of mesoscale eddy kinetic energy decline with measurements of turbulent dissipation. Mesoscale eddies are found to decay at a rate of 0.016 ± 0.012 GW and 0.023 ± 0.017 GW for anticyclonic and cyclonic eddies, respectively, similar to the observed turbulent dissipation rate of 0.020 ± 0.011 GW. This demonstrates that a major direct transfer of mesoscale eddy kinetic energy to small, dissipative scales can be effectively triggered by the eddies’ interaction with the western boundary topography.

## Introduction

Mesoscale eddies—swirling oceanic flows with characteristic horizontal scales of tens to hundreds of kilometres—are ubiquitous in the ocean^1^ and play a fundamental role in the global circulation. As well as accounting for almost 80% of all oceanic kinetic energy^2^, mesoscale eddies effect substantial transports of momentum, heat, carbon and other tracers, thereby shaping the ocean’s large-scale circulation and properties in a number of climatically important ways^3–8^. Determining the processes responsible for the eddies’ generation and dissipation is thus essential to understand, and realistically model, the governing factors of ocean circulation and its climatic impacts. However, large uncertainties persist regarding the mechanisms of eddy dissipation, linked to a general dearth of observations of candidate dissipative processes^9^.

One potentially major mechanism for mesoscale eddy dissipation was highlighted by Zhai et al.^10^, who showed that the western boundaries of ocean basins act as sinks of mesoscale eddy kinetic energy as detected by satellite altimetry. The surface nature of altimetric data, though, prevented these authors from identifying the eddy kinetic energy’s fate, i.e. whether it is largely returned to the large-scale circulation (an *inverse* energy cascade) or dissipated via small-scale turbulence (a *direct* energy cascade). Subsequent work by a range of authors^11–15^ has used theory and idealised numerical simulations to illustrate the dynamical plausibility of a direct cascade pathway to eddy dissipation at western boundaries. Yet, to date, observational evidence of this pathway’s occurrence is lacking within the western boundary eddy kinetic energy sinks.

Here, we address this evidence gap by analysing recent observations of the impingement of three mesoscale eddies (two anticyclonic and one cyclonic) onto the steep and rough topographic slope to the east of of the Bahamian island of Great Abaco^16^—a prominent western-boundary sink of eddy kinetic energy documented by satellite altimetry^10^. The observations were acquired under the auspices of the MeRMEED (Mechanisms Responsible for Mesoscale Eddy Energy Dissipation) project, and included vessel- and mooring-mounted acoustic Doppler current profiler (ADCP) measurements of eddy flows and vertical microstructure profiler- (VMP) based estimates of the turbulent energy dissipation rate across each eddy’s shoreward edge (see Methods). This data set revealed elevated levels of turbulent dissipation above the topographic slope that were especially high for anticyclonic eddies^16^ and occurred in association with a host of eddy-topography interaction processes^16,17^. Such association qualitatively supports the proposition^10^ that western boundaries of ocean basins may be important foci of eddy kinetic energy dissipation. However, a rigorous test of this hypothesis requires that a quantitative assessment of the energetics of the boundary-impinging eddies be performed.

To conduct this assessment, we compare the rate of decay in the energy of mesoscale eddies entering the MeRMEED study domain with the rate of energy dissipation by small-scale turbulence linked to the eddies’ interaction with the local topographic slope. Eddy kinetic energy decay rates are estimated using satellite altimetric measurements of surface geostrophic velocity and mooring-based observations of the eddies’ vertical structure. These are contrasted with energy dissipation rates estimated from VMP measurements, extended and integrated regionally by binning VMP profile data with respect to water depth. We find that, within our study area, mesoscale eddy kinetic energy decays at a pace that closely matches the rate of energy dissipation by small-scale turbulence. This is consistent with eddy-topography interactions underpinning the eddies’ demise via a direct energy cascade, and endorses the view of western boundaries as hotspots of eddy dissipation.

## Mechanisms of mesoscale eddy dissipation

**Figure 1:**
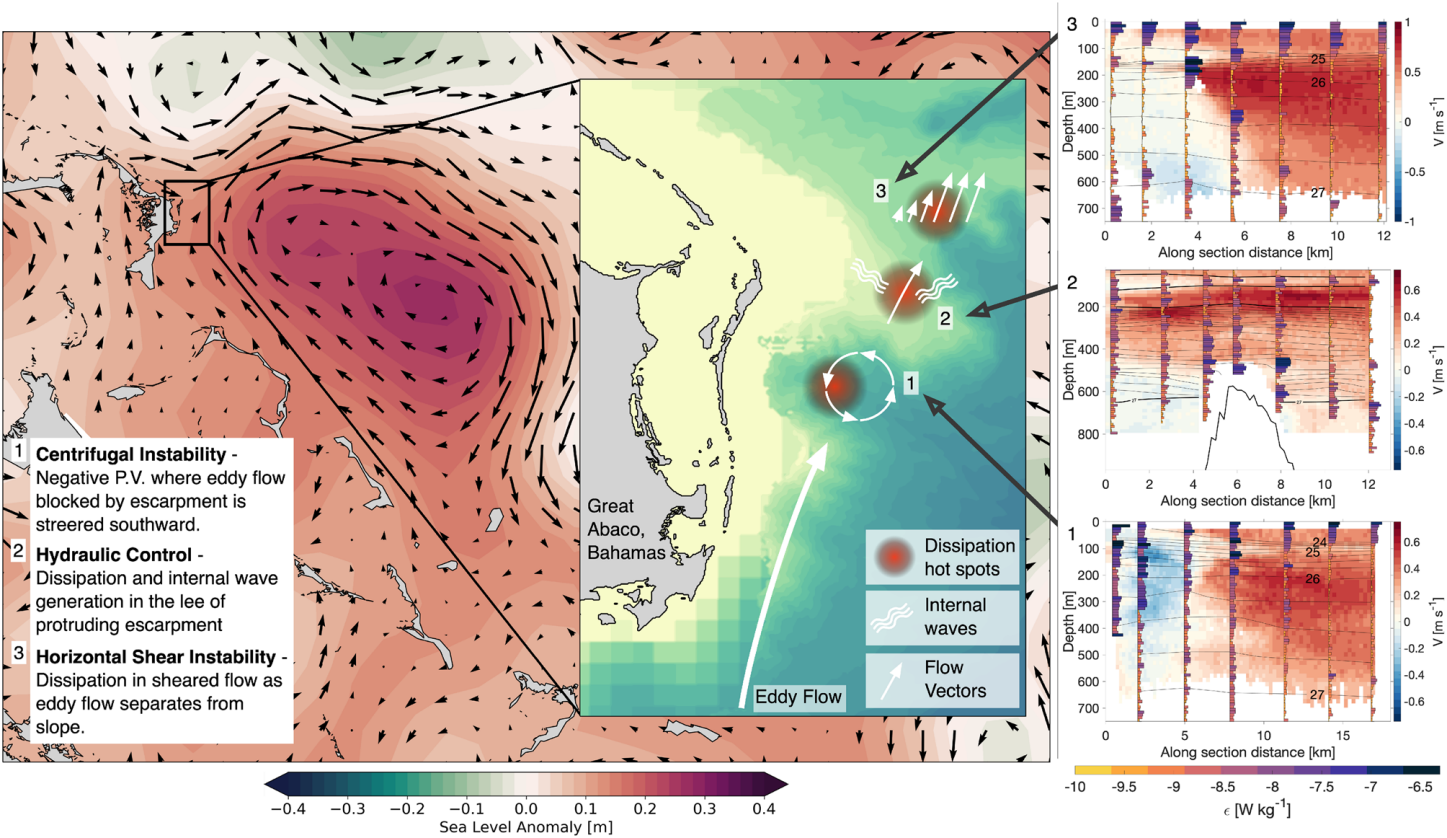
Eddy flow topography interactions. A schematic representation of the varied interactions between the flow of an anticyclonic eddy and the steep and rough slope offshore of Great Abaco, Bahamas. The large panel shows the sea level anomaly (SLA; contours) from October 31 2017 and associated surface geostrophic flow (black arrows). The positive SLA anomaly is an anticyclonic eddy impinging on the eastern slope of the Lucayan Archipelago. The inset panel shows the bathymetry (yellow–blue contours) for a region where the MeRMEED fieldwork took place, and highlights the key regions of eddy flow-topography interactions: (1) Generation of centrifugal instability; (2) Hydraulic control; (3) Generation of horizontal shear instability. The three right hand panels show vessel mounted acoustic Doppler current profiler-based meridional velocity (V) and density (black contours) in each of the three key regions. The stacked bar plots show vertical microstructure-based estimates of the turbulent dissipation in 10 m bins. Each are scaled by $10^{-10}\,\hbox {W}\,\hbox {kg}^{-1}$, and the colour represents the dissipation rate with a logarithmic scale. For more details see Evans et al.^16^. The maps were created using the Python packages cartopy v0.18 and matplotlib v3.3.4, using coastline data from the Global Self-consistent Hierarchical High-resolution Geography (GSHHG; v2.3.7 https://www.ngdc.noaa.gov/mgg/shorelines/) and a compbination of ETOPO1 and multibeam-based bathymetry.

In an observational based study that unravelled the ways in which mesoscale eddies interact with topography in the MeRMEED study region, Evans et al.^16^ highlighted the occurrence of elevated turbulence where a steep and rough topographic slope affects the northward flow of anticyclonic eddies. Their study analysed a section of the slope offshore of the Bahamian island of Great Abaco, in which eddy flow encounters a sloping escarpment that protrudes into the flow, as summarised in Fig. 1.

Both upstream and downstream of this escarpment, turbulence was elevated where the interaction between the mesoscale eddy flow and the topography generated a host of submesoscale processes. Due to the sloping nature of the escarpment, some of the eddy flow is able to pass over the escarpment, while some is blocked. This blocked portion of the eddy flow is steered southward, remaining on the upstream side of the escarpment and recirculating within an indentation of the slope (Fig. 1, example 1). Here, the development of anticylonic vorticity results in a negative potential vorticity (PV) anomaly which, in conjunction with vigorous turbulent dissipation, indicates the presence of centrifugal instability acting to restore PV toward zero. Where the eddy flow passes over the sloping escarpment, elevated Froude numbers, upward-propagating internal waves and near-bottom hot spots of turbulence in the lee of the escarpment, suggest the occurrence of hydraulic control (Fig. 1, example 2).

Downstream of the escarpment, the eddy flow separates from the slope. However, the horizontal and vertical shear imparted on the flow by the slope persists, producing a sloping band of high shear between the eddy flow and the surrounding water (Fig. 1, example 3). In the area of maximum horizontal shear, where turbulent dissipation is also highest, a local change in the isopycnal gradient of PV points to horizontal shear instability as a source of the elevated turbulent dissipation. Further downstream, this strong shear is gradually eroded, reducing the maximum velocity and reinstating stable conditions.

These observations, which synthesise the interaction between an anticyclonic eddy and a steep and rough topographic slope, provide the mechanistic basis for our examination of a western ocean boundary’s potential role as an eddy kinetic energy sink. The analysis of Evans et al.^16^ suggests that when the eddy flow interacts with a topographic slope, the turbulence generated via submesoscale processes can act to dissipate eddy kinetic energy via a direct cascade of energy. Here, we build on this process understanding by quantitatively showing that the decay of eddy kinetic energy in the region closely matches the turbulent dissipation rate. This supports the notion that eddy decay at ocean western boundaries with steep and rough topographic slopes is substantially associated with a direct cascade of energy.

## Results

### Mesoscale eddy decay offshore of the Bahamas

We commence our assessment of mesoscale eddy energetics in the MeRMEED study region by tracking the sources, propagation pathways and sinks of the eddies entering the area, using satellite altimetric observations. In their quasi-global quantification of eddy kinetic energy sources and sinks, Zhai et al.^10^ estimated that the MeRMEED domain hosts a decay of eddy kinetic energy at an approximate rate of 5 mW m$^{-2}$ (per $2^\circ \times 2^\circ$ box), in line with many other western boundary regions world-wide. Close to the MeRMEED study region, this is equivalent to an eddy kinetic energy sink of $\sim 0.2$ GW.

**Figure 2:**
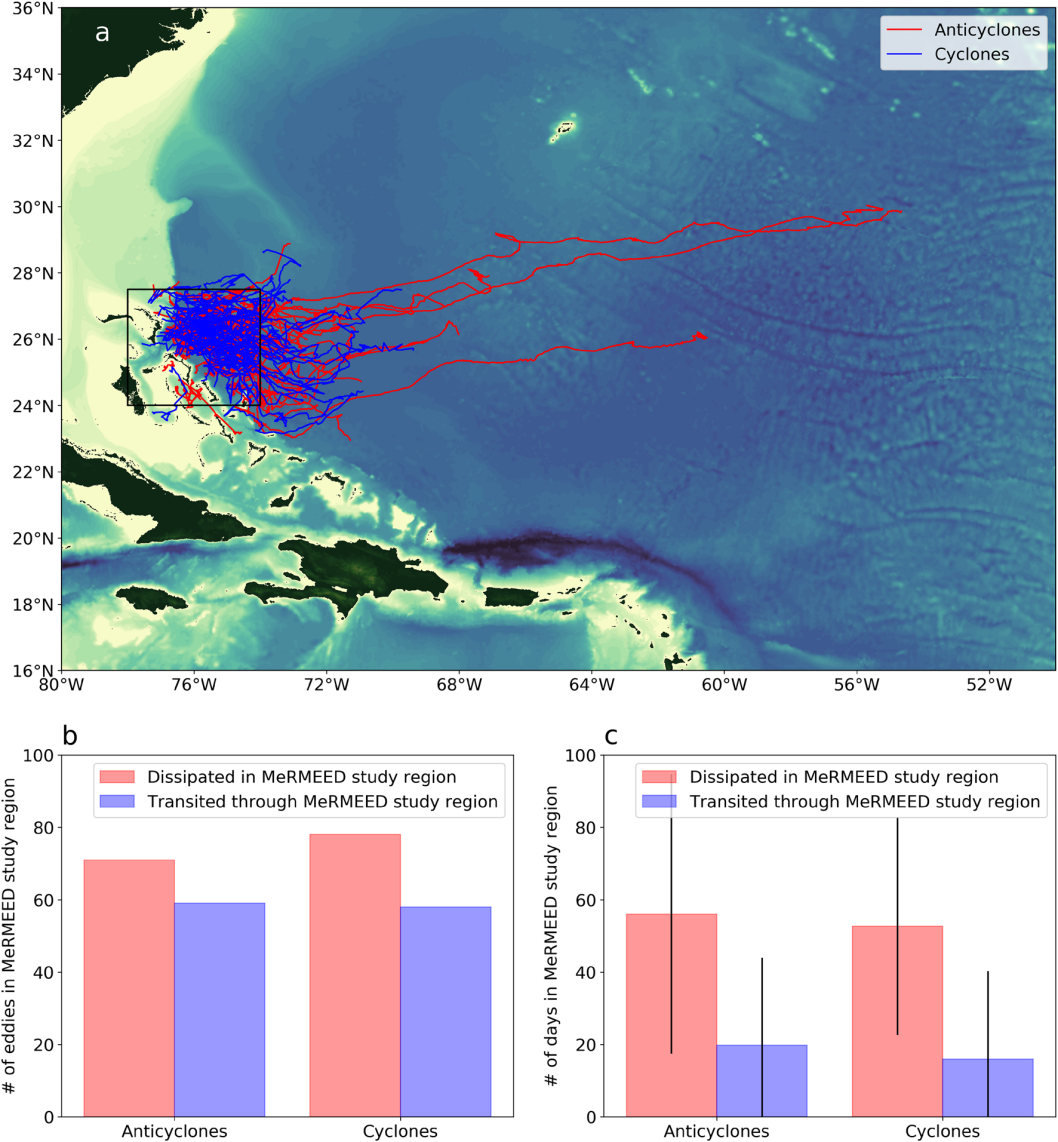
Eddy trajectories and statistics in the MeRMEED study region. (**a**) Trajectories of anticyclonic (red) and cyclonic (blue) eddies that enter the MeRMEED study region marked by the black box during their lifespan between 1993 and 2018. (**b**) The number of anticyclonic (left) and cyclonic (right) eddies that dissipate within the MeRMEED study region (red bar), and that transit through the MeRMEED study region (blue bar). (**c**) The average number of days spent in the MeRMEED study region for those anticyclonic (left) and cyclonic (right) eddies that either dissipate in the MeRMEED study (red bar) region or transit through the MeRMEED study region (blue bar). The vertical black lines represent the one standard deviation about the mean. The map in panel (**a**) was created using the Python packages cartopy v0.18 and matplotlib v3.3.4, and ETOPO1 bathymetry.

Mesoscale eddies that enter the MeRMEED study region (defined as 74$^\circ$ W–78$^\circ$ W, 24$^\circ$ N–27.5$^\circ$ N) are typically formed in the western subtropical North Atlantic, in the zonal band of 70$^\circ$ W–75$^\circ$ W (Fig. 2a). The MeRMEED domain sits adjacent to the steep and rough topographic slope offshore of the Bahamian islands. Due to the lateral curvature of this topographic slope, eddies entering the region typically become trapped against the slope and are prevented from moving meridionally. Anticyclonic eddies may form further to the east than cyclonic eddies, with, for example, two anticyclonic eddies originating at 56$^\circ$ W and a third at 60$^\circ$ W. The origin of mesoscale eddies in Fig. 2 coincides with a source region of eddy kinetic energy identified in previous work^10^.

The total numbers of anticyclonic and cyclonic eddies propagating into the MeRMEED study region are similar, with 130 anticyclonic and 136 cyclonic eddies between 1993 and 2018 (Fig. 2b). The majority of these eddies (71 anticyclonic and 78 cyclonic eddies) remain within the MeRMEED domain until they decay and are no longer detectable in satellite altimetric measurements. The rest of the eddies (59 anticyclonic and 58 cyclonic eddies) leave our study region before decaying. On average, the eddies that decay within the MerMEED domain spend more time in this area ($56\pm 39$ days and $53\pm 30$ days for anticyclonic and cyclonic eddies, respectively) than the eddies decaying elsewhere (Fig. 2c). The latter class of eddies reside in the MeRMEED study region for only $20\pm 24$ days (for anticyclonic eddies) and $16\pm 24$ days (for cyclonic eddies). Here, we report the standard deviation about the mean to highlight the substantial variability in eddy residence times within the MeRMEED domain.

### Subsurface mesoscale eddy structure

The above altimetric view of mesoscale eddy decay in the MeRMEED study region (Fig. 2) enables us to determine the evolving location, horizontal size and surface geostrophic velocity of the eddies sampled by our vessel-based campaigns, which targeted the observation of dissipative processes at each eddy’s onshore edge. However, assessment of the net energy loss implicated in the decay of each eddy requires that the eddy’s vertical structure be known too (see Methods).

**Figure 3:**
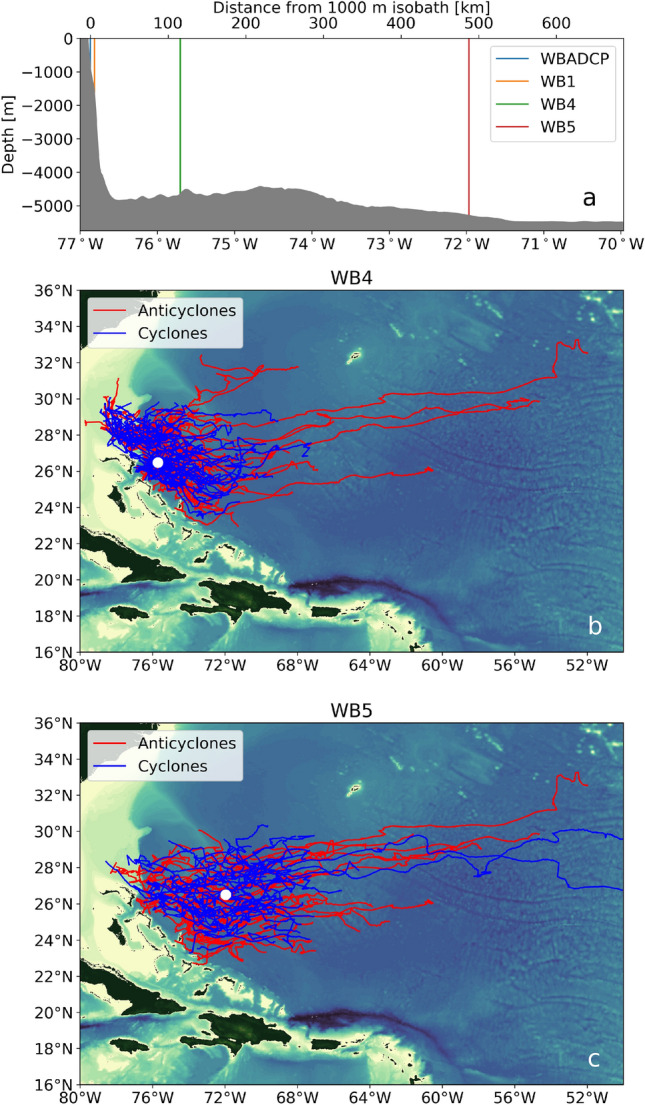
Mesoscale eddies at the RAPID/MOCHA moorings. (**a**) Location of the western boundary RAPID/MOCHA moorings offshore of the Bahamas. The gray shaded area shows the bathymetry. (**b**) Trajectories of anticyclonic (red) and cyclonic (blue) eddies that pass within two eddy radii of the RAPID/MOCHA mooring WB4 during the mooring deployment (2008–2018). (**c**) As in (**b**) but for the RAPID/MOCHA mooring WB5 during the mooring deployment (2004–2014). The maps in panel (**b**,**c**) were created using the Python packages cartopy v0.18 and matplotlib v3.3.4, and ETOPO1 bathymetry.

To constrain this depth dependence, we extrapolate each eddy’s altimetry-based surface geostrophic velocity field in the vertical, by assuming that the eddy’s subsurface velocity follows a first-baroclinic mode structure^18,19^. The validity of this assumption may be illustrated by comparing our estimated eddy velocity profiles with corresponding profiles of geostrophic velocity derived from the RAPID/MOCHA^20^ (Rapid Climate Change / Meridional Overturning Circulation and Heat flux Array) moorings WB4 and WB5. These moorings are located approximately 100 km and 500 km offshore of the Bahamian island of Great Abaco, respectively (Fig. 3a).

**Figure 4:**
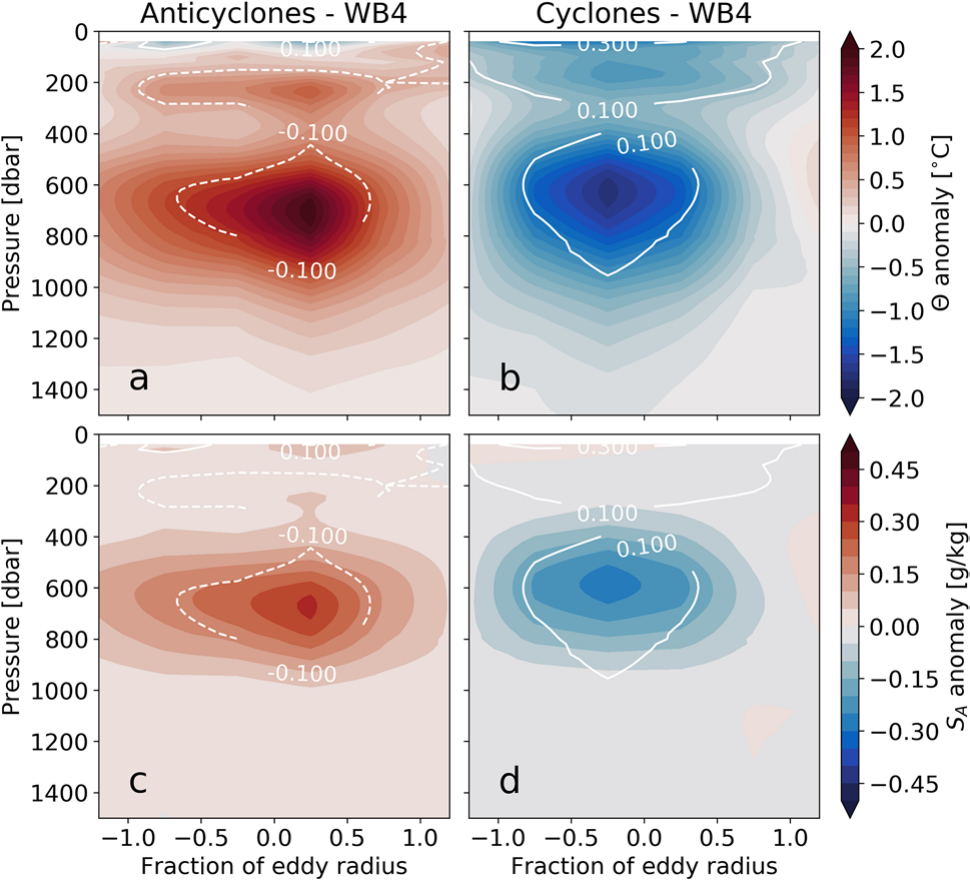
Mesoscale eddy properties at the RAPID/MOCHA mooring WB4. (**a**) Mean $\Theta$ anomaly of anticyclonic eddies that pass RAPID/MOCHA mooring WB4. Mooring observations of $\Theta$ anomaly are binned according to the distance (normalised with respect to the eddy radius) of the eddy from the mooring. The value at a given eddy radius fraction is therefore the mean of all eddies that passed within that fraction of the eddy radius. White contours show $\rho$ anomaly (units kg m$^{-3}$). (**b**) As in (**a**) but for cyclonic eddies. (**c**) As in (**a**) but showing mean $S_A$ anomaly. (**d**) As in (**b**) but showing mean $S_A$ anomaly.

Over the mooring deployment period, 142 and 122 eddies respectively passed within two eddy radii of WB4 and WB5 (Fig. 3b,c). For each of these eddies, we bin the mooring-based observations of conservative temperature anomaly ($\Theta ^{\prime }$), absolute salinity anomaly ($S_A^{\prime }$), potential density ($\rho$) and potential density anomaly ($\rho ^{\prime }$) as a function of eddy radial distance (see Methods). This allows us to build a composite eddy section of $\Theta ^{\prime }$, $S_A^{\prime }$ $\rho$ and $\rho ^{\prime }$ from the moorings for anticyclonic eddies, and another for cyclonic eddies (Fig. 4). We only show eddy sections from WB4, as WB5 shows very similar structure. Anticyclonic (cyclonic) eddies are associated with positive (negative) $\Theta ^{\prime }$ and $S_A^{\prime }$, and negative (positive) $\rho ^{\prime }$. $\rho ^{\prime }$ is largest at the eddy core and typically declines to zero by one eddy radius. This is indicative of the plunging (uplift) of isopycnal surfaces in the core of anticylonic (cyclonic) eddies^21^.

**Figure 5:**
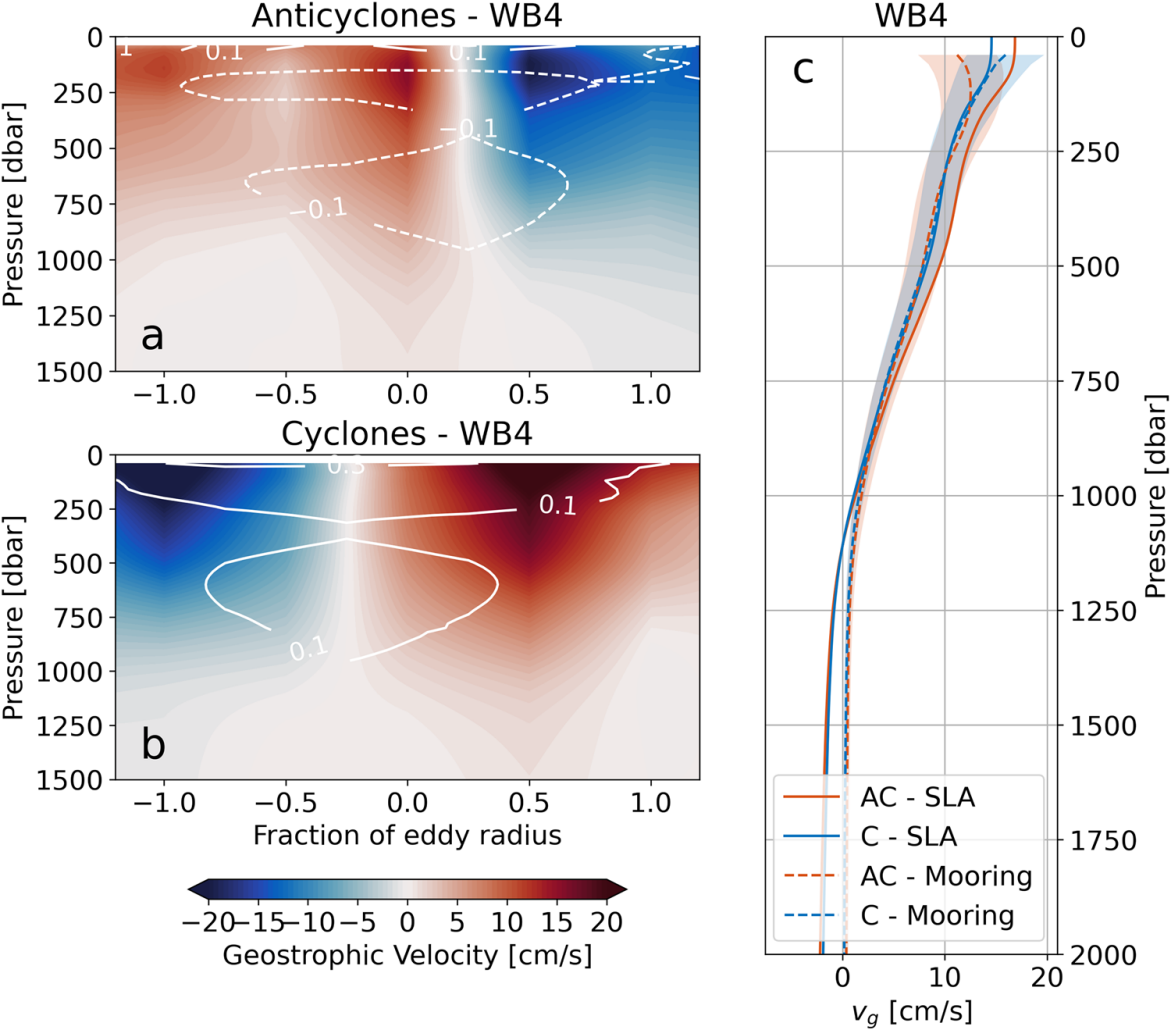
Mesoscale eddy geostrophic velocity at the RAPID/MOCHA mooring WB4. (**a**) Meridional geostrophic velocity calculated using the composite density ($\rho$) sections from the RAPID/MOCHA mooring WB4 for anticyclonic eddies. White contours show density anomaly (($\rho ^{\prime }$) units kg m$^{-3}$). (**b**) As in (**a**) but for cyclonic eddies. (**c**) A comparison between geostrophic velocity at the RAPID/MOCHA mooring WB4 (dashed lines) and the velocity profiles estimated from surface geostrophic velocity derived from altimetry data, assuming sub-surface first mode baroclinic structure (solid lines) for anticyclonic (orange) and cyclonic (blue) eddies. The shading indicates an uncertainty range for the mooring-based estimate using Monte-Carlo based bootstrapping to calculate upper and lower bounds for density.

The composite sections of $\rho$ are used to compute geostrophic velocity normal to the sections, for anticyclonic (Fig. 5a) and cyclonic (Fig. 5b) eddies. These highlight the rotational sense of the respective eddies, which are clockwise and anticlockwise in the northern hemisphere. Mean profiles of absolute geostrophic velocity from WB4 provide a point of comparison for our extrapolated, altimetry-based surface geostrophic velocity profiles (Fig. 5c). For both anticyclonic and cyclonic eddies, the mooring-based and altimetry-based velocity profiles agree within the estimated error of the mooring-based profiles at depths shallower than 1000 m. Below 1000 m, the mooring-based profile asymptotes to zero, whereas our altimetry-based velocity is closer to -2 cm s$^{-1}$ according to the veritical structure of the first baroclinic mode. As a result, we restrict our calculation of eddy kinetic energy to the uppermost 1000 m, which is the depth range where eddy flow is typically strongest and where we observe the most intense turbulent dissipation along the topographic slope (see section 4 in Evans et al.^16^). All in all, this analysis demonstrates that our assumption of a first-mode baroclinic structure in vertically extrapolating altimetry-based surface geostrophic velocities, holds for the top 1000 m of both anticyclonic and cyclonic eddies.

### Mesoscale eddy kinetic energy decay versus turbulent dissipation above topography

**Figure 6:**
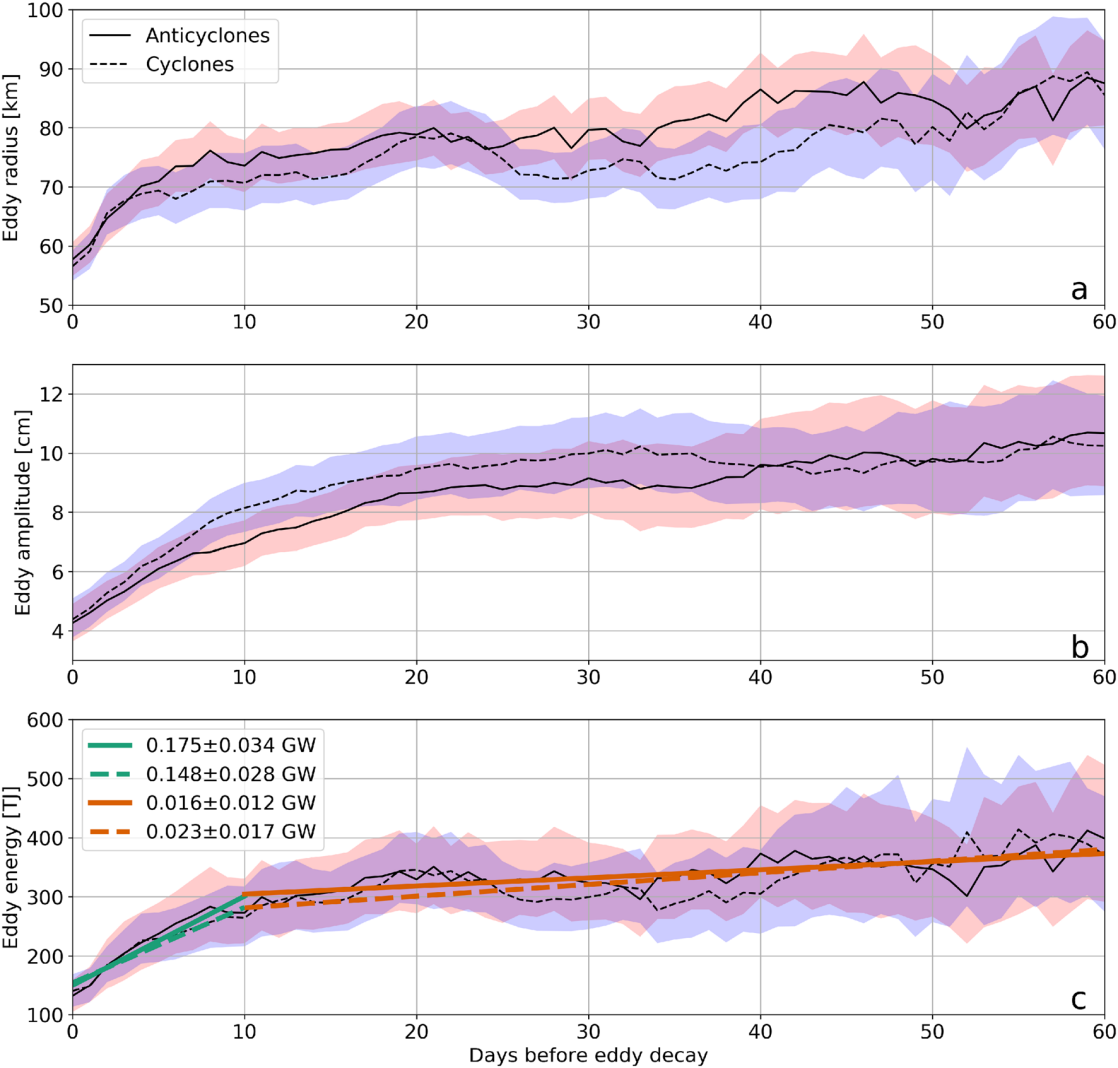
Mesoscale eddy decay rate. (**a**) Mean eddy radius of anticyclonic (red) and cyclonic (blue) eddies that dissipate within the MerMEED study region, shown as a function of days before decay. The shading represents an estimate of the uncertainty in the eddy radius calculated using Monte-Carlo based bootstrapping. (**b**) As in (**a**) but for eddy amplitude. (**c**) As in (**a**) but for eddy kinetic energy. The coloured lines are a linear fit to the eddy kinetic energy curves for the intervals 0–10 days before decay and 10–60 days before decay. The gradient of each coloured line is shown in the legend where the uncertainty range is estimated from the mean difference between the gradient of the upper and lower bounds (represented by the shading) and the central curve.

We next compute the rate of kinetic energy decay for each of the mesoscale eddies dying off in the MeRMEED study region (Fig. 2a). To enable this calculation, we track the change in eddy radius (Fig. 6a) and amplitude (Fig. 6b), from which we estimate eddy kinetic energy ($E_{eddy}$; Fig. 6c) by: (i) vertically extrapolating the eddy’s altimetry-based surface geostrophic velocity with a first-mode baroclinic structure (Fig. 5c); and (ii) integrating the resulting velocity profiles with depth and over the surface area of the eddy. See Methods for a full description of the eddy kinetic energy calculation procedure. Note that we consider solely an eddy’s kinetic energy and not its total energy, which includes the much larger reservoir of available potential energy^2^. We focus on changes in an eddy’s kinetic energy because, unlike those in the eddy’s potential energy, they can be directly connected to irreversible turbulent dissipation^2,9,22^. See Methods for a detailed discussion of the eddy kinetic energy equation, and the assumptions underpinning our comparison of $E_{eddy}$ decay versus turbulent dissipation. Results are shown in Fig. 6a–c, where each line represents an average of (anticyclonic or cyclonic) eddies as a function of days before decay in the MeRMEED domain. Averages are over $\sim$30 eddies at 60 days before decay, increasing to 70–75 eddies at 0–30 days before decay.

Both eddy radius and amplitude decrease steadily from 60 to 10 days before decay, with the rate of decay increasing in the final 10 days before the eddy disappears. The rates of decline of eddy radius and amplitude are similar for anticyclonic and cyclonic eddies. These patterns of change hold for $E_{eddy}$ too, linking the decline of eddy radius and amplitude to the decline in eddy kinetic energy. During 60-10 days before decay, $E_{eddy}$ decreases at a rate of 0.016 ± 0.012 GW for anticyclonic eddies and 0.023 ± 0.017 GW for cyclonic eddies. The decay rate intensifies to 0.175 ± 0.034 GW and 0.148 ± 0.028 GW, respectively, between 10 and 0 days. While the latter pair of decay rates are closer to our approximation of the eddy sink reported in Zhai et al.^10^, it is likely that decay rates during 10–0 days before decay are biased high, as the eddy diameter drops below the resolution of altimetric data ($\sim$30 km). We therefore concentrate on the period between 60 and 10 days for our comparison between rates of eddy kinetic energy decay and turbulent dissipation above the topographic slope.

**Figure 7:**
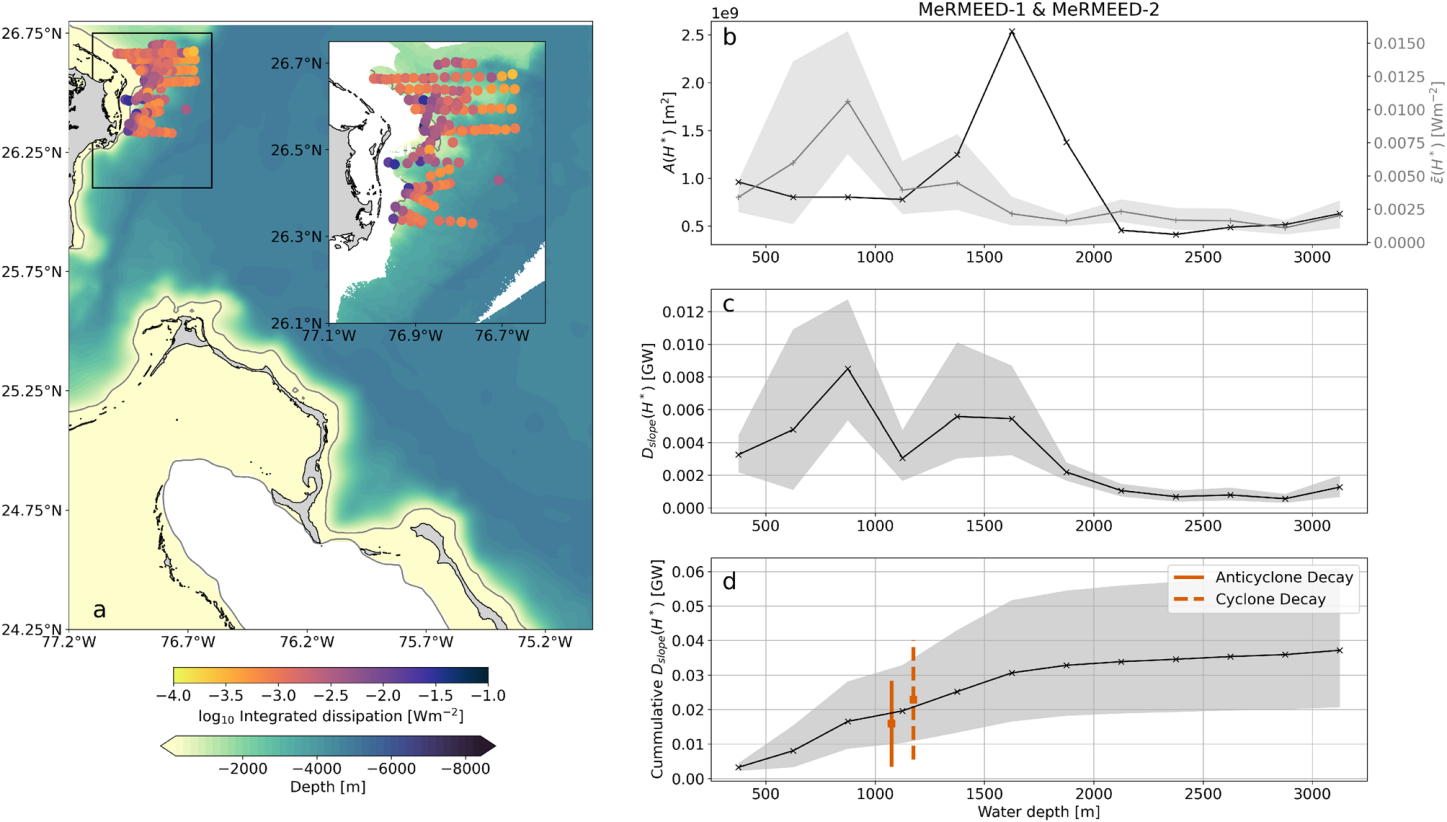
Turbulent dissipation in the MeRMEED study region. (**a**) Integrated turbulent dissipation measured using a tethered vertical microstructure profiler during the MeRMEED fieldwork campaign. Bathymetery from ETOPO-1 is shown in the main panel and swath-based bathymetry is shown in the inset panel that shows the MeRMEED cruise region. The grey contour is the 250 m depth contour. (**b**) Total isobath area ($A(H^*)$) for bins of water depth based on ETOPO-1 for the entire domain shown in (**a**) and integrated isobath dissipation rate ($\bar{\epsilon }(H^*)$) averaged in depth bins based on the depth at the location of the profile. (**c**) Turbulent dissipation rate ($D_{slope}(H^*)$) from the product of $A(H^*)$ and $\bar{\epsilon }(H^*)$. (**d**) $D_{slope}(H^*)$ accumulated in depth space shown with the eddy kinetic energy decay rate for anticyclones (orange solid line) cyclones (orange dashed line) between 10 and 60 days before eddy decay from Fig. 6c. The shading represents an estimate of the uncertainty in the mean isobath integrated dissipation rate calculated using Monte-Carlo based bootstrapping. The map in panel (**a**) was created using the Python packages cartopy v0.18 and matplotlib v3.3.4, with ETOPO1 bathymetry and GSHHG coastline data.

The spatial patterns of turbulent dissipation in the MeRMEED study region during the impingement of the sampled eddies on the western boundary (see Methods) are illustrated in Fig. 7, which displays the VMP-measured, vertically-integrated rate of turbulent dissipation. Elevated dissipation is widespread above the topographic slope, and is most intense in regions shallower than 1000 m where eddies interact with the steep and rough bathymetry offshore of Great Abaco. Evans et al.^16^ showed that the strong dissipation above the slope is primarily underpinned by a range of processes triggered by the eddies’ flow over the corrugated topography of the boundary, namely: submesoscale centrifugal and horizontal shear instabilities, hydraulic control, and the radiation and breaking of internal waves (Fig. 1). Thus, the bulk of the vigorous turbulence apparent in Fig. 7 is expected to act to dissipate the impinging eddies.

The area sampled by the MeRMEED cruises (inset panel in Fig. 7a) focused on a relatively small section of the topographic slope, compared to the size of a typical mesoscale eddy at this latitude (encompassed by the MeRMEED study region, which is indicated by the black box in Figure 2a). Thus, determining the fraction of the rate of eddy kinetic energy decline that is accounted for by turbulent dissipation requires that the cruise-based measurements of dissipation be extended to the entire topographic slope within the MeRMEED study region. With this purpose, we bin our observations of vertically-integrated dissipation rate into water depth (*H*) bins, giving $\bar{\epsilon }(H^*)$ (Fig. 7c). The rate of eddy dissipation via interactions with the topographic slope in the MeRMEED study region, $D_{slope}(H^*)$, can then be assessed by multiplying $\bar{\epsilon }(H^*)$ by the area of each water depth bin ($A(H^*)$). We calculate $A(H^*)$ for a domain that is slightly smaller than the full MeRMEED study region, to omit parts of this region with topography distinct from that in the area of the cruises (e.g., to the north and west of the MeRMEED study region). The ETOPO-1 bathymetric data used to calculate $A(H^*)$ is spanned by the domain shown in the larger panel of Figure 7a.

Within the MeRMEED study region, $\bar{\epsilon }(H^*)$ is largest where water depth is between 750 m and 1000 m, reaching 0.010 ± 0.005 W m$^{-2}$ (Fig. 7c). This rate of turbulent dissipation is slightly larger than the eddy decay rate of $\sim$0.005 W m$^{-2}$ reported by Zhai et al.^10^. At water depths shallower and deeper than 750-1000 m, $\bar{\epsilon }(H^*)$ decreases quickly, down to a minimum of 0.001 ± 0.0005 W m$^{-2}$ over water depths between 2750 m and 3000 m. The distribution of $A(H^*)$ indicates that a relatively large surface area and shallow slope characterise isobaths between 1250 m and 1500 m, with a broadly constant slope at other water depths (Fig. 7b). $D_{slope}(H^*)$ is highest between 750 m and 1000 m at 0.009 ± 0.004 GW, and generally adopts modest values at water depths exceeding 1250 m. Thus, the dissipative action of turbulence on the eddies’ onshore edge is most vigorous in the upper part of the slope, in water depths shallower than 1250 m, where eddy flows are most intense (Fig. 5). The accumulation of $D_{slope}(H^*)$ with respect to water depth readily demonstrates this fact, as it increases to 0.020 ± 0.011 GW between 1000 and 1250 m, and plateaus at water depths greater than 1500 m (Fig. 7d).

Our estimate of $D_{slope}(H^*)$ is an approximation of the energy lost by eddies impinging on the western boundary of the MeRMEED study region to smaller scales, via a direct cascade underpinned by flow-topography interactions^16^. We can now compare $D_{slope}(H^*)$ to the rate of energy decay for eddies entering the MeRMEED domain. This comparison reveals that the rates of decay of $E_{eddy}$, for both anticyclonic and cyclonic eddies, agree within error with $D_{slope}(H^*)$ for depths shallower than 1250 m (Fig. 7d). This suggests that the decay of mesoscale eddies in the MeRMEED study region can be largely accounted for by turbulent dissipation triggered by eddy flow-topography interactions. These interactions result in a direct cascade of energy from the mesoscale to the small scales of three-dimensional turbulence, at which eddy kinetic energy irreversibly dissipates.

## Discussion and conclusions

We have shown that the decay of mesoscale eddy kinetic energy in a region offshore of the Bahamian islands, typical of the western boundary of the North Atlantic^10^, is driven predominantly by the dissipative action of small-scale turbulence, which is generated by the impingement of eddy flows onto the boundary’s steep and rough topographic slope. Our demonstration rests on the favourable comparison between regional eddy kinetic energy decay rates estimated from satellite altimetric and mooring observations, and ship-based measurements of turbulent dissipation rates associated with eddy-topography interactions. In the 60-10 day period before disappearing from altimetry, eddies decaying in our study region do so at rates of 0.016 ± 0.012 GW (for anticyclonic eddies) and 0.023 ± 0.017 GW (for cyclonic eddies). Similarly, the eddy flow-topography interactions reported along the boundary^16^ dissipate energy at a rate of 0.020 ± 0.011 GW in water depths shallower than 1250 m, where eddy flows are largest.

To make this comparison, we adopt two key assumptions. The first assumption relates to the subsurface configuration of eddy flow, which we reconstruct from altimetry-based surface geostrophic velocity with a first-mode baroclinic structure. We demonstrate that this assumption is valid for depths shallower than 1000 m, by comparing the reconstructed velocity to profiles of geostrophic flow at the RAPID/MOCHA moorings WB4 and WB5. The second assumption entails the extrapolation of VMP-based estimates of vertically-integrated turbulent dissipation via binning with respect to water depth, from the region of the field campaign to a larger portion of the western boundary on which a mesoscale eddy would typically impinge. This larger region is defined by selecting a wider segment of the topographic slope with similar steepness and roughness to that of the cruise-based measurements, and with a meridional extent matching the characteristic scale of a mesoscale eddy at the latitude of our study. Eddies in this larger region are typically prevented from moving meridionally, due to the substantial lateral curvature of the slope. This assumption is justified by the focussing of intense turbulent dissipation within a very narrow range of water depth bins, representing the portion of the slope intercepting the eddy flows.

An important potential caveat to our findings concerns the potential occurrence in our study region of small-scale turbulence-generating processes unrelated to mesoscale eddies, such as the breaking of wind-forced near-inertial waves or internal tides. These processes may conceivably elevate turbulent dissipation in the MeRMEED domain, and thereby exaggerate the perceived importance of the eddy dissipation pathway via a direct energy cascade. However, available evidence suggests that turbulent dissipation in our study region is weak in the absence of eddy flow-topography interactions. Thus, the elevated dissipation rates observed during our field campaign were highly localised to areas of eddy flow impingement on topography, and were readily linked to specific submesoscale processes^16^. Further, Clément et al.^17^ showed that the dissipation rate in the MeRMEED area was reduced as much as four-fold in the local absence of eddies, or when a cyclonic eddy was present. This result was confirmed by the third cruise of the MeRMEED fieldwork campaign, which took place during a small cyclonic eddy that remained away from the slope (see Methods for details). The dissipation rates ($D_{slope}$) measured in this cruise were approximately half of those observed during the initial two MeRMEED cruises, used in the present analysis.

Previous studies have broadly quantified the size of the mesoscale eddy sink along oceanic western boundaries^10^. Our estimates of mesoscale eddy kinetic energy decay agree with these studies. However, no previous work has been able to provide observational evidence that the eddies’ decay at western boundaries is underpinned by turbulent dissipation, rather than by an inverse energy transfer to the large-scale ocean circulation. Our results suggest that the direct cascade of energy from the mesoscale to the small scales of three-dimensional turbulence is an important pathway for the irreversible dissipation of the ocean’s mesoscale eddy field.

The widespread impingement of mesoscale eddies on steep and rough topography along other oceanic western boundaries, as well as on island chains, points to the likely role of the direct energy cascade highlighted in this work as an important sink in the global ocean energy budget. In our study region, elevated turbulent dissipation is primarily associated with the interaction of eddy flows with corrugations in the topographic slope. Where an eddy flow impinges on corrugated bathymetry, a range of dissipative flow-topography interactions results, including submesoscale centrifugal and horizontal shear instabilities, hydraulic control, and internal wave radiation and breaking^16,17^. Thus, in order to credibly represent the mesoscale eddy field and its wider climatic influence, numerical models must sufficiently resolve these flow-topography interactions, or include appropriate parameterisation. For example, the representation of the meridional overturning circulation in ocean models is acutely sensitive to the way in which the eddies’ dissipation is parameterised, as such dissipation impacts the modelled western-boundary flow and its associated meridional transports of mass and heat^23–29^. Our results indicate that the models’ spatial resolution may be critical to the realistic representation of the eddies’ damping, as the most intense dissipation takes place within 10-20 km of the coast and is underpinned by physical processes with respective horizontal and vertical scales of O(1 km) and O(10 m). We thus conclude that capturing the dissipative effects of these processes stands out as an important challenge for the next generation of ocean models.

## Methods

In this study, we estimate the decay rate of mesoscale eddy kinetic energy in a region offshore of the Bahamian islands in the tropical North West Atlantic (MeRMEED study region: 74$^\circ$ W–78$^\circ$ W, 24$^\circ$ N–27.5$^\circ$ N, chosen to capture a portion of the topographic slope large enough to affect an entire eddy). To quantify eddy kinetic energy decay, we use satellite-based estimates of surface geostrophic velocity, and extrapolate them vertically by assuming that surface velocities are indicative of a first-mode baroclinic structure below the surface. We then track the changes in eddy kinetic energy within our study region to estimate a decay rate. We compare this decay rate to the turbulent dissipation rate measured during a fieldwork campaign that took place along a portion of this domain using a tethered vertical microstructure profiler (VMP). In the following section, we outline the data used in our analysis, the methods and assumptions adopted to estimate eddy kinetic energy and decay rate, and our approach for extrapolating the VMP-based observations to the wider MeRMEED study region in order to robustly compare turbulent dissipation and eddy decay rate estimates.

### Data and processing

The work described in this study forms part of the MeRMEED (Mechanisms Responsible for Mesoscale Eddy Energy Dissipation) project. A portion of the MeRMEED fieldwork involved three ship-based VMP surveys to measure the tubulent dissipation rate offshore of Great Abaco, Bahamas (MeRMEED cruise region: 77.1$^\circ$W–76.6$^\circ$W, 26.1$^\circ$N–26.75$^\circ$N). The outcome of this survey and other aspects of the MeRMEED project are reported in Evans et al.^16^, and also in Fernández Castro et al.^30^. The VMP data used in the present study was collected over the course of three separate cruises that sampled two different anticyclonic eddies and one (likely) cyclonic eddy, respectively: MeRMEED-1 (1–7 December 2016), MeRMEED-2 (31 October–10 November 2017) and MeRMEED-3 (4–16 March 2018). As reported in Evans et al.^16^, the eddy conditions during MeRMEED-3 were somewhat uncertain. This uncertainty resulted from a discrepancy between how the eddy was resolved in satellite altimetric observations, compared to satellite-based, higher-resolution sea surface temperature data. The sea surface temperature data indicated that the cyclonic eddy, which appeared adjacent to the slope in altimetry, may not have been intercepted by our near-boundary measurements. As a result, our analysis in this study focuses on the observations made during MeRMEED-1 and MeRMEED-2. VMP profiles were typically performed along zonal sections that ran from on-slope at water depths of approximately 400 m, to 10–15 km offshore in water depths exceeding 4000 m, with an along-section resolution of approximately 500 m. Details of VMP processing can be found within Evans et al.^16^. All the MeRMEED data can be accessed through the British Oceanographic Data Centre (Moored ADCP: https://doi.org/10/fjpx, MeRMEED-1: https://doi.org/10/fjp7, MeRMEED-2: https://doi.org/10/fjqh, MeRMEED-3: https://doi.org/10/fjq2).

We compare the VMP-based estimates of turbulent dissipation rates to estimates of mesoscale eddy kinetic energy decay rates derived from satellite-based sea level data. These data were accessed via the Copernicus Marine Environment Monitoring Service (https://marine.copernicus.eu/). We use the daily reprocessed multi-mission global ocean gridded L4 product for sea surface height and surface geostrophic velocity for the period 1993–2019. The data have a horizontal resolution of 0.25 degrees. To complement these data, we also use an atlas for mesocale eddy trajectories derived from sea level observations produced by SSALTO/DUACS and distributed by AVISO+ (https://www.aviso.altimetry.fr/) with support from CNES, developed and validated in collaboration with D. Chelton and M. Schlax at the Oregon State University. This atlas spans the entire satellite altimetry period and provides trajectories, amplitudes and radii for individual anticyclonic and cyclonic eddies. This allows eddies that enter and dissipate within the MeRMEED study region to be tracked throughout their lifespan in the sea level data set.

To define the structure of the first baroclinic mode in the MeRMEED study region, we use vertical profiles of temperature and salinity from the western portion of the A05 GO-SHIP hydrographic section, to calculate the buoyancy frequency, $N^2$, a measure of the vertical stratification. We select all available profiles between 65$^\circ$W and 70$^\circ$ from the 2004^31^, 2010^32^ and 2015^33^ occupations of A05, accessed via https://cchdo.ucsd.edu/. To calculate $N^2$, we adiabatically sort fluid parcels according to the methodology outlined in Bray and Fofonoff^34^, and interpolate the resultant profiles of $N^2$ onto a regular 2 dbar grid. To estimate the vertical structure of the first baroclinic mode, we then apply a normal mode decomposition^35^, deriving normal modes of horizontal velocity from $N^2$.

We validate our estimate of the subsurface velocity field, and our assumption of a first-mode baroclinic structure, using mooring-based profiles of temperature and salinity from the RAPID/MOCHA^20^ (Rapid Climate Change / Meridional Overturning Circulation and Heat flux Array) moorings WB4 and WB5. The data are provided with a 20 dbar vertical resolution and a 12 hour temporal resolution. Data were accessed at http://www.rapid.ac.uk/rapidmoc/. WB4 data spans 2008–2018, and WB5 data spans 2004–2014. We use TEOS-10^36^ to calculate conservative temperature ($\Theta$), absolute salinity ($S_A$) and potential density ($\rho$). We further calculate $\Theta$ anomaly ($\Theta ^{\prime }$), $S_A$ anomaly ($S_A^{\prime }$) and $\rho$ anomaly ($\rho ^{\prime }$) with respect to the time mean for each mooring.

Using the mesoscale eddy trajectory atlas, we build a composite cross section of $\Theta ^{\prime }$, $S_A^{\prime }$, $\rho$ and $\rho ^{\prime }$ during anticyclonic and cyclonic eddies that pass the moorings. Eddies passing near the moorings are identified using a distance metric between the fixed mooring position and the trajectory of the eddy centre from the mesoscale eddy trajectory atlas. We select segments of the trajectory that are within $-1.25 \le r \le 1.25$ of the mooring position, based on the eddy radius (*r*) according to the mesoscale eddy trajectory atlas. Using the mooring-based profiles of $\Theta ^{\prime }$, $S_A^{\prime }$, $\rho$ and $\rho ^{\prime }$ from each time point of the trajectory segment, we ascribe a fractional radius (distance from mooring divided by *r*) to that profile of $\Theta ^{\prime }$, $S_A^{\prime }$, $\rho$ and $\rho ^{\prime }$. Based on this fractional radius, the mooring-based $\Theta ^{\prime }$, $S_A^{\prime }$, $\rho$ and $\rho ^{\prime }$ profiles are averaged within bins of fractional radius from the eddy centre from $-1.25$ to 1.25 at intervals of $\Delta r = 0.5$. From the composite section of $\rho$, we then compute meridional geostrophic velocity following the thermal wind relation.

A total of 11 anticyclonic and 16 cyclonic eddies passed within $\pm 0.25$ of an eddy radius of WB4, we therefore had to use relatively large bins of eddy radius at $\Delta r = 0.5$. This allowed us to represent the large scale structure of eddies at WB4, but as a consequence of a large $\Delta r$, the zero velocity does not fall exactly at the eddy centre. However, this does not affect our comparison to the altimetry-based eddy velocity profile, which uses the mean magnitude of the velocity. For detailed bathymetry within the MeRMEED cruise region, we use multibeam-based data acquired from the National Centers for Environmental Information (https://www.ncei.noaa.gov/). For bathymetry over the larger MeRMEED study region, we use ETOPO1, a 1 arc-minute global relief model^37^.

### Estimating mesoscale eddy kinetic energy decay rate

To quantify the mesoscale eddy kinetic energy decay rate, we use altimetry-based estimates of surface geostrophic velocity over the area of an eddy, where the eddy radius is extracted from the eddy tracking atlas. For each individual eddy that dissipates within the MeRMEED study region, we find values of the surface zonal and meridional geostrophic velocity components that fall within the radius of the eddy, at each day within its life-span in the altimetric record. We extrapolate these velocities vertically, assuming that surface flows are representative of a first-mode baroclinic structure below the surface. The kinetic energy of a given eddy is therefore:

1 $$\begin{aligned} E_{eddy}(t) = \int \int \Pi (d\le r)\left[ \frac{\rho _0}{2}\int _{-1000}^{0} u'(x,y,z,t)^2+v'(x,y,z,t)^2 \,\mathrm {d}z\right] \,\mathrm {d}x\,\mathrm {d}y , \end{aligned}$$

where $u'$ and $v'$ denote the three-dimensional fields of the eddy’s zonal and meridional velocity components, respectively, $\rho _0=1025$ kg m$^{-3}$ is the background density, and $\Pi$ is a boxcar function that is either 1 when the distance from the eddy centre *d* is within the eddy radius *r*, or otherwise 0. This gives the time-varying eddy kinetic energy, $E_{eddy}$, in units of Joules.

$E_{eddy}$ is linked to the rate of turbulent dissipation, $\epsilon$, through the eddy kinetic energy equation^22,38^:

2 $$\begin{aligned} \frac{\partial \frac{1}{2}(\overline{u'^2} + \overline{v'^2})}{\partial t} \; = \; -\frac{\partial (\frac{1}{2}\overline{\mathrm {u}_j}\overline{\mathrm {u}_i'^2} + \frac{1}{2}\overline{\mathrm {u}_j'\mathrm {u}_i'^2} + \frac{1}{\rho _0}\overline{\mathrm {u}'_j p'})}{\partial x_j} \; + \; -\overline{\mathrm {u}'_j \mathrm {u}'_i}\frac{\partial \overline{\mathrm {u}_i}}{\partial x_j} \; + \; \overline{w'b'} \; + \; \epsilon , \end{aligned}$$

where $i=1,2$ and $j=1,2,3$ following Cartesian tensor notation with summation convention, so that $\mathrm {u}_1=u$, $\mathrm {u}_2 = v$ and $\mathrm {u}_3=w$. The overline represents a time mean, and primes indicate anomalies with respect to that time mean. Further, *p* denotes the pressure anomaly, and $b=-\frac{g\rho }{\rho _0}$ is the buoyancy. The first term on the right hand side of (2) is the transport of eddy kinetic energy, the second term is the conversion of mean kinetic energy to eddy kinetic energy, and the third term is the conversion of eddy potential energy to eddy kinetic energy. $\epsilon$ amalgamates the contributions to eddy kinetic energy dissipation from the vertical mixing and horizontal diffusion terms in the horizontal momentum equation. In this study, we assume that $\epsilon$ provides the primary sink of $E_{eddy}$ in (2). This assumption is grounded on a range of preceding works. For example, Nikurashin et al.^39^ showed, using high-resolution numerical simulations of a Southern Ocean region, that turbulent dissipation acts as the dominant sink of the kinetic energy of geostrophic flows over rough topography. Similarly, Yang et al.^22^ and Zhang et al.^40^ indicated that mesoscale eddies encountering rough topography in the South China Sea are predominantly dissipated by small-scale turbulent processes. In comparing the dissipation term to the conversion terms in (2) in numerical simulations spanning a variety of flow and topographic regimes, Yang et al.^22^, Gula et al.^38^ and Zhang et al.^40^ all find $\epsilon$ to greatly exceed the conversion terms. Thus, in our work, we focus on the investigation of $\epsilon$ as the main sink of eddy kinetic energy.

### Extrapolating VMP-based measurements of turbulent dissipation

To extrapolate our VMP-based profiles of $\epsilon$ from the smaller MeRMEED cruise area to the larger MeRMEED study region, we bin our observations according to water depth. This assumes that the turbulent dissipation rate is centrally linked to the bathymetry of the study region, as is suggested by its fundamental underpinning by eddy flow-topography interactions^16^. Further, the largest dissipation rates are typically observed at depths shallower than 1000 m, where eddy flows are strongest^16^.

We integrate $\epsilon$ vertically between the maximum depth of the profile, $z_{max}$, and 50 m, and calculate the mean integrated dissipation where the depth of the profile *H* is in the range $H^*\pm \Delta H/2$, where $H^*$ is the profile depth at the bin centre and $\Delta H/2$ represents the profile depth bin width:

3 $$\begin{aligned} \bar{\epsilon }(H^*)= \frac{1}{M}\sum \left[ \Pi (H,H^*)\,\rho _0\int _{-z_{max}}^{-50}\epsilon \, \mathrm {d}z\right] . \end{aligned}$$

Here, *M* is the number of VMP profiles per water depth bin, and $\Pi$ is a boxcar function that is either 1 when *H* is in the range $H^*\pm \Delta H/2$ or otherwise 0. The mean integrated dissipation for each water depth bin, $\bar{\epsilon }(H^*)$, is then multiplied by the total area of that bin, $A(H^*)$

4 $$\begin{aligned} A(H^*)=\int \int \Pi (H,H^*)\,\mathrm {d}x\,\mathrm {d}y, \end{aligned}$$

to give a turbulent dissipation rate where $H^*\pm \Delta H/2$:

5 $$\begin{aligned} D_{slope}(H^*)=\bar{\epsilon }(H^*)\times A(H^*). \end{aligned}$$

To conclude, we calculate the turbulent dissipation rate for the MeRMEED study region using ETOPO-1 bathymetry and water depth bins of 250 m, centered at intervals of 375–3125 m.
